# Profiling of euphane and tirucallane triterpenes from *Euphorbia systyloides* on antichagasic activity reveals antileukemia and immunomodulatory effects

**DOI:** 10.1080/13880209.2026.2722390

**Published:** 2026-08-28

**Authors:** Mohamed Abakar Maaz, Anita Barta, Dragana Markovic, Simon Singer, Jürg Gertsch, Róbert Berkecz, Peter Waweru Mwangi, Judit Hohmann, Andrea Vasas

**Affiliations:** a Institute of Pharmacognosy, University of Szeged, Szeged, Hungary; b HUN-REN-USZ Biologically Active Natural Products Research Group, University of Szeged, Szeged, Hungary; c Institute of Biochemistry and Molecular Medicine, University of Bern, Bern, Switzerland; d Institute of Pharmaceutical Analysis, University of Szeged, Szeged, Hungary; e School of Biological Sciences, University of Nairobi, Nairobi, Kenya

**Keywords:** *Euphorbia systyloides*, triterpenes, *Trypanosoma cruzi*, antileukemic, immunomodulatory effect

## Abstract

**Background:**

*Euphorbia* is one of the largest genera in the spurge family and is rich in bioactive secondary metabolites, especially triterpenoids and diterpenoids. *Euphorbia systyloides* Pax. (Euphorbiaceae) is native to tropical Africa, and its leaf decoction has traditionally been used to treat tapeworm infections. Its chemistry has not been previously investigated.

**Objective:**

This study aimed to investigate the terpenoid constituents of *E. systyloides* and evaluate the bioactivity of the compounds against *Trypanosoma cruzi* and tumor cells.

**Materials and methods:**

Compounds were isolated by multistep chromatography and structurally elucidated using HRMS and 1D/2D NMR. Biological activity was assessed against *T. cruzi* epimastigotes (XTT assay), and host cell infection and amastigote replication assays, while cytotoxicity was evaluated in HL60, U937, and PBMCs (MTT assay). Immunomodulatory effects were assessed by ELISA measurement of TNF-α and IL-10 in infected macrophages.

**Results:**

Six new euphane and tirucallane triterpenes and six known triterpenes were isolated. None were selectively active against *T. cruzi*. One ergostane triterpene exhibited nanomolar cytotoxicity against HL60 and U937 cell lines, with moderate toxicity toward PBMCs. Unexpectedly, kansenonol enhanced amastigote replication in host cells, likely through macrophage immunomodulation.

**Discussion and conclusions:**

Our results indicate that the ergostane triterpene represents an unexplored scaffold for antileukemic drug discovery. Based on an unexpected observation in a cellular *T. cruzi* trypomastigote infection assay, kansenonol was found to exert significant immunomodulatory effects *in vitro*. Overall, this study shows that triterpenoids from *E. systyloides* primarily exert cytotoxic and immunomodulatory effects rather than selective antiparasitic activity.

## Introduction

*Euphorbia* is one of the largest genera in the spurge family (Euphorbiaceae) (Mavundza et al. 2022). Many species in this genus are widely distributed across semiarid, subtropical, and tropical regions and are utilized for medicinal, ecological, and economic purposes (Bruyns et al. 2011; Anest et al. 2021). Species of the genus *Euphorbia* are characterized by producing latex, a milky exudate containing a diverse array of bioactive secondary metabolites. Among these, terpenoids – particularly di- and triterpenes – are the most abundant classes of compounds (Benjamaa et al. 2022; Zhao et al. 2022; Kgosiemang et al. 2025). Other compounds, such as phenolics (e.g., flavonoids), alkaloids, and saponins, are also present in the species (Tostes et al. 2019; Douglas et al. 2021). The biological activities of the plants are mainly attributed to their terpenoid content (Shi et al. 2008).

*Euphorbia* species have been extensively used in traditional medicine for centuries to treat skin irritations, wounds, and digestive disorders (Amtaghri et al. 2022; Chafik et al. 2023). Additionally, *Euphorbia* plants have been used to treat inflammations, tumors, gout, back pain, arthritis, pneumonia, and gonorrhea (Nouri et al. 2017). The reported pharmacological effects of *Euphorbia* species encompass anti-inflammatory, cytotoxic, anticancer, antibacterial, antifungal, and antiviral properties (Tsai et al. 2021; Oliveira et al. 2022; Mishra and Parida 2023; Rojas-Jiménez et al. 2024).

Chagas disease is a serious, life-threatening, neglected tropical disease caused by the protozoan parasite *Trypanosoma cruzi,* and is also known as American trypanosomiasis. The disease was first discovered in Latin America, where it exhibited high mortality rates; it is defined as a chronic infectious condition associated with poverty because it affects socially vulnerable individuals, leading to considerable physical, psychological, and socioeconomic burdens (Messenger et al. 2015; Malik et al. 2025). In the search for antichagasic molecules, investigating the selective toxicity and effectiveness of plant natural products against the infective form of *T. cruzi* is a feasible approach (Izumi et al. 2011; Salm et al. 2021).

**Figure 1.**
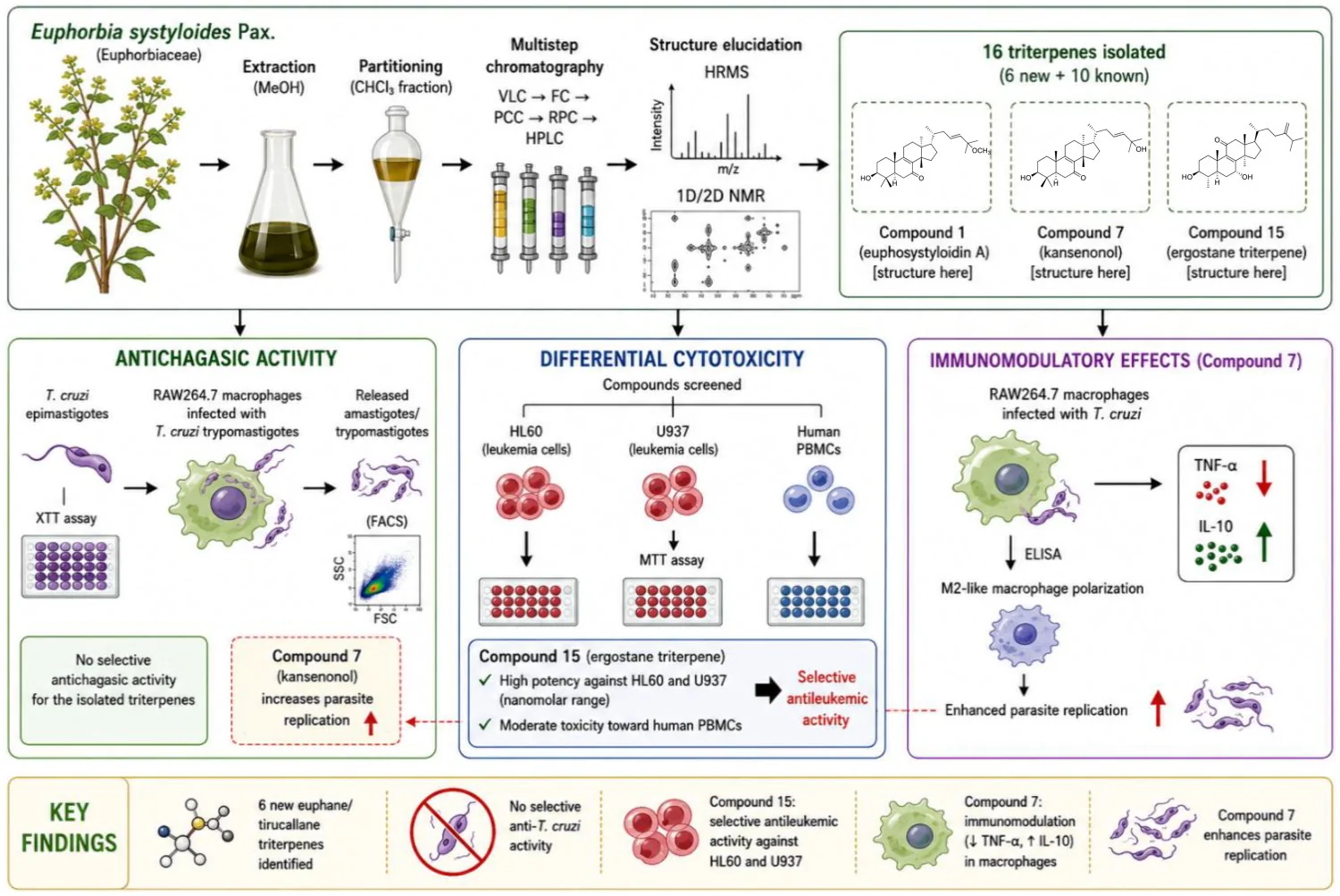
Illustration of the workflow for the phytochemical and biological evaluation of *E. systyloides* triterpenes. Image was generated using BioRender and Google Gemini 3 Pro.

*Euphorbia systyloides* Pax. (syn. *Euphorbia holstii*, *E. volkensii*) (Euphorbiaceae) is an herbaceous annual plant belonging to the Euphorbiaceae family, and usually grows up to 1.50 m. The plant is native to Kenya, Angola, Uganda, and other tropical countries in southern Africa. Building on our previous findings on the selective antichagasic effects of triterpenoids (Saidu et al. 2024), we aimed to investigate the anti-*Trypanosoma cruzi* activity of triterpenes isolated from *E. systyloides*. Although information on *E. systyloides* is limited, a leaf decoction of the plant is reportedly prepared with salt to treat tapeworm infections (tropical.theferns.info 2026). This article presents the first investigation of *E. systyloides* phytoconstituents and provides the initial profiling of the isolated cytotoxic triterpenoids. Based on an unexpected observation in the cellular *T. cruzi* trypomastigote infection assay, we show that the euphane-type triterpenoid kansenonol, first isolated in 2003 from *E. kansui* (Wang et al. 2003), exerts significant immunomodulatory effects *in vitro* (Figure 1).

**Figure 2.**
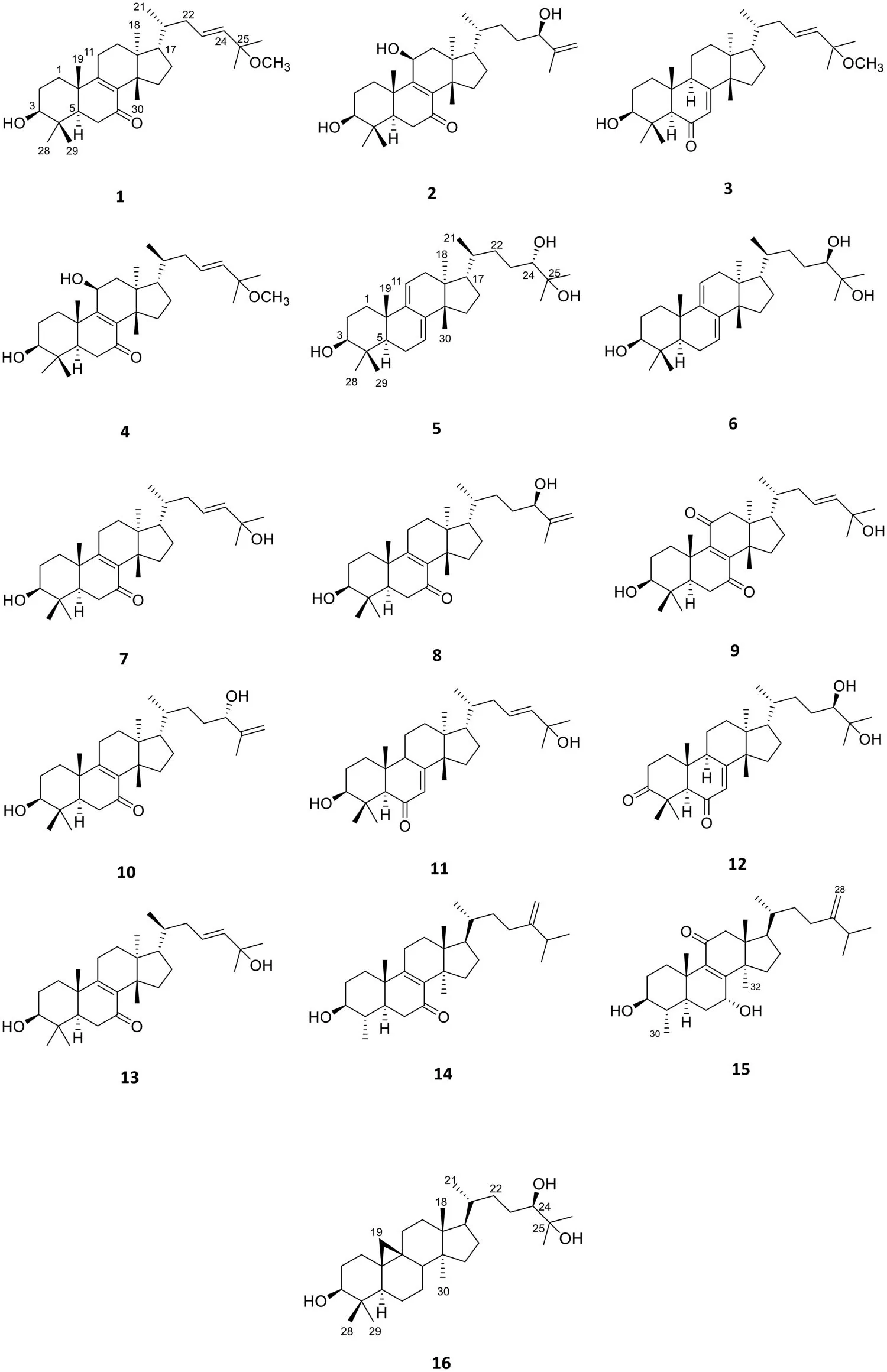
Structure of the isolated compounds **1–16**.

## Materials and methods

### General experimental procedures

Optical rotation measurements were conducted using a Jasco P-2000 polarimeter (Jasco International Co. Ltd., Hachioji, Tokyo, Japan). NMR spectra were recorded in CD_3_OD or CDCl_3_ on a Bruker Avance DRX 500 spectrometer at 500 MHz (^1^H) and 125 MHz (^13^C), with the residual nondeuterated solvent signals as internal references. HRESIMS was performed on a Thermo Scientific Q-Exactive Plus Orbitrap (Thermo Fisher Scientific Co. Ltd., USA) mass spectrometer using either an ESI or an APCI ion source in positive ionization mode. The MassLynx software was used to acquire and process data. Vacuum liquid chromatography (VLC) was performed to separate the extract and fractions using silica gel (15 μm, Merck, Product No. Merck, 1.07730). OCC was performed using polyamide (MP Biomedicals, Product No. 0209602). Furthermore, flash chromatography (FC) was conducted using a CombiFlash NextGen 300 +, EZ Prep instrument (Serial: 221K00745, USA; Teledyne Isco) equipped with integrated ultraviolet (UV), UV − visible (UV − Vis), and evaporative light scattering detection employing manually assembled columns with normal-phase (NP; silica gel, 15 *μ*m; Merck) and reversed-phase (RP; LiChroprep RP-18, 40 − 63 μm; Merck) stationary phases. Preparative Thin-Layer Chromatography (PTLC) was performed using silica gel 60 F_254_ plates (20 × 20 cm silica gel 60 F_254_, Product No. Merck 1055540001). Rotational planar chromatography (RPC) was carried out using a Harrison Research Chromatotron centrifugal thin-layer chromatograph (Serial No. HR5248, USA). High-Performance Liquid Chromatography (HPLC) was performed using Shimadzu HPLC instruments equipped with a DGU-403 degassing unit, an LC-40D solvent delivery module, an SPD-M40 photodiode array detector, and a RP Kinetex C18 100 A (4.6 mm × 150 mm, 5 *μ*m, Product No. 00 F-4601-E0) analytical column. Extracts were dried using a rotavapor (Büchi, R-220 SE and R-210, Switzerland), equipped with a vacuum pump (Büchi, V-100). TLC chromatograms were visualized under UV light at 254 nm, then sprayed with a vanillin-concentrated sulfuric acid mixture, and heated for 1 min at 110 °C. Solvents used for the extraction, OCC, VLC, FC, and PTLC were of analytical grade supplied by Molar Chemicals Kft. (Halásztelek, Hungary): methanol (Product No. 05730-006-410), *n*-hexane (Product No. 06850-101-411) chloroform (Product No. 04730-101-411), ethyl acetate (Product No. 02930-101-411), etanol (Product No. 02911-101-340), and acetonitril (Product No. 06850-101-411). Solvents for HPLC were purchased from VWR International Kft. (Debrecen, Hungary): methanol (Product No. 20864.320), and acetonitril (Product No. 20060.320). The water was supplied by the Milli-Q Direct 3 UV pump (Merck, Darmstadt, Germany).

### Plant material

*E. systyloides* Pax. aerial parts were collected, dried, and ground in Kenya in 2021 (GPS coordinates: −1.6899520, 37.3630470); they were authenticated and identified by Peter Waweru Mwangi (Department of Medical Physiology, School of Medicine, University of Nairobi, Nairobi, Kenya). A voucher specimen (No. PMWUON2020/003) has been deposited in the herbarium of the Department of Medical Physiology, School of Medicine, University of Nairobi, Nairobi, Kenya.

### Extraction and isolation

The dried, ground plant material was percolated with methanol (MeOH). The extract was evaporated to dryness (668.6 g), then dissolved in 50% aqueous MeOH and successively partitioned with *n*-hexane, chloroform, and ethyl acetate (EtOAc). The CHCl_3_ phase was subjected to VLC using a gradient solvent system of cyclohexane–EtOAc (from 98:2 to 40:60), yielding 11 fractions (Fr. 1–11).

Fractions (Frs. 9 and 10) were further separated by RP-FC using a Teledyne ISCO C18 silica gel (24 g) RediSep manually packed flash column with a MeOH–H_2_O gradient system at a flow rate of 35 mL/min. Fractions 9/5 and 9/8 were then subjected to polyamide column chromatography (PCC) using MeOH–H_2_O gradient elution (3:2, 4:2, and 1:0) to obtain Frs 9/5/1–3 and 9/8/1–3. Subsequently, Fr. 9/5/1 was separated by preparative RP-HPLC (Armen–Spot Prep II system) on a Gemini NX-C18 column (110 Å, 5 µm, 250 × 21.2 mm, Phenomenex, Torrance, CA, USA, Product No. 00 G-4454-P0-AX) using a MeOH–H_2_O gradient elution. The obtained fractions were finally purified by analytical RP-HPLC using different isocratic ratios of MeOH–H_2_O to yield compounds **6** (8:2, 2.49 mg, *t*_R_ = 21.2 min) and **5** (8:2, 2.15 mg, *t*_R_ = 22.5 min), compound **13** (7:3, 2.10 mg, *t*_R_ = 19.9 min), compounds **9** (68:32, 1.17 mg, *t*_R_ = 19.7 min) and **4** (68:32, 1.64 mg, *t*_R_ = 23.8 min), and compound **16** (79:21, 0.7 mg, *t*_R_ = 22.0 min); acetonitrile–H_2_O (57:43) was used for compounds **7** (1.76 mg, *t*_R_ = 8.4 min), **8** (1.64 mg, *t*_R_ = 9.4 min), **10** (2.56 mg, *t*_R_ = 9.9 min), and **11** (2.67 mg, *t*_R_ = 10.7 min).

Fraction 10 was first separated by PCC using a MeOH–H_2_O gradient elution with ratios of 3:2, 4:2, and 1:0 to obtain Fr. 10/1–3, followed by NP-FC using an *n*-hexane–acetone gradient system for Fr. 10/1 to yield 18 subfractions (Fr. 10/1/1–18). Then, RP-FC separation was performed on Frs 10/1/12 and 13. The obtained subfractions were finally purified by RP-HPLC using a solvent system of acetonitrile–H_2_O (6:4) to yield compound **2** (2.10 mg, *t*_R_  =  23.1 min), and MeOH–H_2_O (69:31) to yield compound **12** (1.64 mg, *t*_R_  =  11.6 min).

Compounds **1** (3.43 mg, *t*_R_  =  17.4 min), **3** (3.09 mg, *t*_R_  =  19.3 min), **15** (3.71 mg, *t*_R_  =  12.9 min) and **14** (1.38 mg, *t*_R_  =  16.1 min) were isolated from Fr. 8 *via* three successive isolation steps: an RP-FC using a MeOH–H_2_O gradient system (from 10% to 100% MeOH), followed by RPC using a cyclohexane–EtOAc gradient system (from 9:1 to 4:6), and finally cyclohexane–EtOAc–MeOH (6:3:1) in the last step. The pure components were finally obtained by RP-HPLC using MeOH–H_2_O in ratios of 23:77 for compounds **1** and **3**, and 20:80 and 15:85 for compounds **15** and **14**, respectively.

*Euphosystyloidin A* (**1**): colorless amorphous material; [*α*]_D_^26^ +19.5 (*c* 0.3, MeOH); ^1^H and ^13^C NMR data, see Tables 1 and 2; HR-APCI-MS + at *m/z* 471.3827 [M + H]^+^ (calcd for C_31_H_51_O_3_^+^: 471.3833), 439.3565 [M + H –CH_3_OH]^+^ (calcd for C_30_H_47_O_2_^+^: 439.3571).
*Euphosystyloidin B* (**2**): a white amorphous solid; [*α*]_D_^26^ +0.4 (*c* 0.3, MeOH); ^1^H and ^13^C NMR data, see Tables 1 and 2; HRESIMS + *m/z* 455.3517 [M + H – H_2_O]^+^ (calcd for C_30_H_47_O_3_^+^: 455.3525), 437.3412 [M + H –2H_2_O] (calcd for C_30_H_45_O_2_^+^: 437.3414).
*Euphosystyloidin C* (**3**): a white amorphous solid; [*α*]_D_^26^ +7.7 (*c* 0.2, MeOH); ^1^H and ^13^C NMR data, see Tables 1 and 2; HRESIMS *m/z* 471.3826 [M + H]^+^ (calcd for C_31_H_51_O_3_^+^: 471.3833), 439.3568 [M + H – CH_3_OH]^+^ (calcd for C_30_H_47_O_2_^+^: 439.3571).
*Euphosystyloidin D* (**4**): a white amorphous solid; [*α*]_D_^26^ –2.2 (*c* 0.2, MeOH); ^1^H and ^13^C NMR data, see Tables 2 and 3; HRESIMS *m/z* 487.3765 [M + H]^+^ (calculated for C_31_H_51_O_4_^+^: 487.3787), 455.3514 [M + H – CH_3_OH]^+^ (calculated for C_30_H_47_O_3_^+^: 455.3520), 437.3410 [M + H – CH_3_OH – H_2_O]^+^ (calculated for C_30_H_45_O_2_^+^: 437.3410).
*Euphosystyloidin E* (**5**): a white amorphous substance; [*α*]_D_^26^ –66.7 (*c* 0.1, MeOH); ^1^H and ^13^C NMR data, see Tables 2 and 3; HRESIMS *m/z* 459.3826 [M + H]^+^ (calculated for C_30_H_51_O_3_^+^: 459.3833), 441.3722 [M + H – H_2_O]^+^ (calculated for C_30_H_49_O_2_^+^: 441.3732).
*Euphosystyloidin F* (**6**): a white amorphous substance; [*α*]_D_^26^ –53.3 (*c* 0.2, MeOH); ^1^H and ^13^C NMR data, see Tables 2 and 3; HRESIMS *m/z* 459.3817 [M + H]^+^ (calculated for C_30_H_51_O_3_^+^: 459.3833).

**Table 1.** ^1^H NMR data of the isolated euphane triterpenes (**1**–**3**, **9**, **10**, **12**) [500 MHz, *δ* ppm (*J*  =  Hz)].

Position	1^a^^,^^c^	2^a^	3^a^^,^^c^	9^b^	10^a^	12^b^
**1**α	1.41, m	1.54, m	1.41, m	1.18, m	1.44, m	1.82, m
**1** β	1.86, m	2.43, m	1.70, m	2.46, m	1.87, m	2.01, m
**2**	1.68 m 1.74 m	1.77, m 1.90, m	1.67, m α 1.59, m β	1.70, m (2H)	1.68, m 1.76, m	2.27, dt (15.2, 3.0) 2.81, dt (15.2, 5.5)
**3**	3.29 br d (9.8)	3.31, dd (13.7, 6.6)	3.22, br d (11.0)	3.24, dd (11.7, 4.4)	3.29, dd (11.8, 3.6)	–
**5**	1.67 dd (13.9, 5.3)	1.65, dd (13.8, 5.0)	2.13, s	1.73, m	1.67, dd (13.0, 5.5)	2.67 s
**6**	2.36 m β 2.44 dd (11.0, 5.3) α	2.44, m (2H)	–	2.51, d (9.8) (2H)	2.39, m, 2.42 m	–
**7**	–	–	5.70, d (1.9)	–	–	5.71, d (2.0)
**9**	–	–	2.72, dd (12.0, 4.2)	–	–	2.96, dd (13.1, 5.1)
**11**	2.24 dt (20.7, 3.9) β 2.37 m α	4.70, t (8.1)	1.58, m 1.73, dd (14.0, 5.1)	–	2.22, m, 2.42 m	1.73, m 1.81, dd (14.0, 5.1)
**12**	1.79 m (2H)	1.81, m β 2.40, dd (12.4, 8.1) α	1.77, m α 1.90, t (12.9) β	2.48, d (19.3) 2.70, d (19.3)	1.79, m (2H)	1.90, m 1.98, m
**15**	1.52, m α 2.13, br t (11.2) β	1.42, m 2.08, m	1.54, m (2H)	1.69, m 2.09, m	1.53, m 2.12, m	1.57, m 1.65, m
**16α**	1.36, m	1.31, m	1.36, m	1.44, m	1.37, m	1.54, m
**16β**	1.94, m	1.93, m	1.99, ddd (16.5, 15.8, 8.8)	2.03, m	1.95, m	2.07, m
**17**	1.53, m	1.60, m	1.58, m	1.76, m	1.52, m	1.65, m
**18**	0.76, s	0.72, s	0.86, s	1.00, s	0.73, s	0.91, s
**19**	1.06, s	1.26, s	0.86, s	1.31, s	1.05, s	1.12, s
**20**	1.55, m	1.47, m	1.50, m	1.56, m	1.51, m	1.49, m
**21**	0.86, d (5.7)	0.87, d (6.8)	0.85, d (7.5)	0.90 d (6.4)	0.88,d (6.5)	0.90, d (6.0)
**22**	1.86, m 2.37, m	1.20, m (2H)	1.73, m 2.36, dt (13.2, 3.8)	1.79, m 2.26, ddd (13.7, 6.1, 2.4)	1.21, m 1.50, m	1.24, m 1.73, m
**23**	5.52 ddd (15.9, 7.8, 7.3)	1.45, m (2H)	5.51, dt (15.6, 7.0)	5.59, m	1.47, m, 1.58 m	1.41, m (2H)
**24**	5.40 d (15.9)	4.03, t (6.6)	5.40, d (15.6)	5.59, m	4.03, t (6.2)	3.26, d (10.1)
**26**	1.25, s	4.86, s 4.95, s	1.26, s	1.27, s	4.85, s 4.94, s	1.14, s
**27**	1.25, s	1.74, s	1.26, s	1.27, s	1.73, s	1.16, s
**28**	1.00, s	1.00, s	1.32, s	1.01, s	1.00, s	1.32, s
**29**	0.90, s	0.92, s	1.13, s	0.90, s	0.89, s	1.34, s
**30**	0.98, s	1.15, s	1.06, s	1.08, s	0.97, s	1.15, s

^a^ Measured in CDCl_3_.

^b^ In CD_3_OD.

^c^ 25-OMe**:** 3.15 s.

**Table 2.** ^13^C NMR data of the isolated compounds **1**–**6**, **9**, **10**, and **12**–**16** (125 MHz, *δ,* ppm).

Position	1^a^	2^a^	3^a^	4^b^	5^b^	6^b^	9^b^	10^a^	12^b^	13^b^	14^b^	15^b^	16^b^
**1**	34.8, CH_2_	33.9, CH_2_	37.1, CH_2_	34.6, CH_2_	37.1, CH_2_	37.3, CH_2_	35.1, CH_2_	34.8, CH_2_	38.2, CH_2_	35.7, CH_2_	35.3, CH_2_	34.8, CH_2_	33.3, CH_2_
**2**	27.6, CH_2_	27.6, CH_2_	26.8, CH_2_	28.5, CH_2_	32.3, CH_2_	32.3, CH_2_	28.0, CH_2_	27.6, CH_2_	35.0, CH_2_	28.1, CH_2_	31.7, CH_2_	31.9, CH_2_	31.1, CH_2_
**3**	78.2, CH	78.5, CH	79.3, CH	78.9, CH	79.7, CH	79.8, CH	78.6, CH	78.3, CH	217.2, C	78.6, CH	75.9, CH	77.1, CH	79.5, CH
**4**	39.0, C	39.2, C	38.2, C	40.1, C	40.1, C	40.1, C	39.6, C	39.0, C	48.2, C	39.9, C	40.1, C	38.7, C	41.6, C
**5**	48.5, CH	49.4, CH	65.4, CH	50.7, CH	49.9, CH	49.9, CH	49.7, CH	48.4, CH	66.0, CH	49.6, CH	48.5, CH	44.0, CH	48.7, CH
**6**	35.9, CH_2_	36.0, CH_2_	200.1, C	36.8, CH_2_	24.8, CH_2_	24.8, CH_2_	36.7, CH_2_	36.0, CH_2_	201.0, C	36.7, CH_2_	40.1, CH_2_	31.2, CH_2_	22.3, CH_2_
**7**	198.3, C	200.1, C	125.2, CH	202.7, C	119.9, CH	119.9, CH	201.7, C	198.4, C	125.2, CH	201.0, C	200.8, C	67.9, CH	27.2, CH_2_
**8**	139.1, C	140.6, C	170.5, C	141.3, C	142.6, C	142.6, C	151.1, C	139.1, C	174.4, C	139.9, C	140.5, C	164.2, C	49.6, CH
**9**	165.3, C	161.2, C	50.5, CH	165.1, C	146.6, C	146.6, C	156.4, C	165.4, C	50.6, CH	169.3, C	168.5, C	141.5, C	21.1, C
**10**	39.5, C	39.7, C	43.9, C	38.5, C	37.3, C	37.1, C	39.3, C	39.4, C	44.5, C	40.6, C	40.3, C	38.8, C	27.4, C
**11**	23.8, CH_2_	68.3, CH	17.7, CH_2_	68.3, CH	116.5, CH	116.6, CH	203.5, C	23.9, CH_2_	18.7, CH_2_	24.9, CH_2_	25.7, CH_2_	203.2, C	27.6, CH_2_
**12**	30.2, CH_2_	43.0, CH_2_	32.9, CH_2_	43.4, CH_2_	39.5, CH_2_	39.7, CH_2_	52.4, CH_2_	30.1, CH_2_	34.0, CH_2_	31.3, CH_2_	31.4, CH_2_	53.1, CH_2_	34.2, CH_2_
**13**	44.9, C	46.4, C	43.2, C	47.5, C	45.3, C	45.3, C	46.4, C	44.8, C	44.2, C	45.9, C	46.2, C	49.0, C	46.4, C
**14**	47.8, C	48.2, C	52.5, C	49.1, C	50.8, C	50.8, C	49.1, C	47.9, C	53.8, C	49.1, C	49.7, C	52.4, C	50.0, C
**15**	31.6, CH_2_	32.0, CH_2_	33.0, CH_2_	33.2, CH_2_	29.2, CH_2_	29.4, CH_2_	32.9, CH_2_	31.2, CH_2_	34.0, CH_2_	32.7, CH_2_	33.3, CH_2_	31.0, CH_2_	36.7, CH_2_
**16**	28.6, CH_2_	28.0, CH_2_	27.9, CH_2_	28.1, CH_2_	28.4, CH_2_	28.4, CH_2_	28.9, CH_2_	28.8, CH_2_	29.0, CH_2_	29.4, CH_2_	29.7, CH_2_	28.2, CH_2_	29.1, CH_2_
**17**	48.3, CH	48.8, CH	52.7, CH	49.7, CH	52.0, CH	52.6, CH	50.0, CH	48.5, CH	54.4, CH	49.1, CH	50.4, CH	51.4, CH	53.8, CH
**18**	16.1, CH_3_	16.5, CH_3_	22.3, CH_3_	16.8, CH_3_	16.8, CH_3_	16.8, CH_3_	19.0, CH_3_	15.9, CH_3_	22.3, CH_3_	16.3, CH_3_	16.3, CH_3_	17.2, CH_3_	18.6, CH_3_
**19**	18.8, CH_3_	19.9, CH_3_	14.4, CH_3_	19.8, CH_3_	21.3, CH_3_	21.3, CH_3_	18.2, CH_3_	19.1, CH_3_	14.2, CH_3_	18.9, CH_3_	18.0, CH_3_	16.4, CH_3_	31.1, CH_2_
**20**	36.1, CH	35.7, CH	36.1, CH	37.1, CH	37.8, CH	37.1, CH	37.3, CH	35.8, CH	36.8, CH	37.3, CH	37.5, CH	37.5, CH	37.8, CH
**21**	19.3, CH_3_	19.0, CH_3_	18.9, CH_3_	19.5, CH_3_	19.5, CH_3_	19.2, CH_3_	19.1, CH_3_	19.1, CH_3_	18.8, CH_3_	19.5, CH_3_	19.2, CH_3_	18.9, CH_3_	19.0, CH_3_
**22**	38.7, CH_2_	31.2, CH_2_	38.4, CH_2_	39.5, CH_2_	33.9, CH_2_	33.4, CH_2_	38.9, CH_2_	31.6, CH_2_	33.2, CH_2_	35.5, CH_2_	36.2, CH_2_	35.9, CH_2_	35.0, CH_2_
**23**	128.7, CH	31.6, CH_2_	128.9, CH	130.6, CH	29.2, CH_2_	29.1, CH_2_	125.7, CH	31.6, CH_2_	29.3, CH_2_	126.1, CH	32.3, CH_2_	32.2, CH_2_	29.2, CH_2_
**24**	136.9, CH	76.3,CH	136.9,,CH	137.3, CH	80.5, CH	79.6, CH	141.1, CH	76.4, CH	79.5, CH	140.8, CH	157.7, C	157.6, C	80.7, CH
**25**	75.0, C	148.0, C	75.0, C	76.5, C	73.9, C	73.9, C	71.2, C	148.0, C	73.9, C	71.0, C	34.9, CH	34.9, CH	73.9, C
**26**	26.0, CH_3_	111.1, CH_2_	25.9, CH_3_	26.3, CH_3_	24.9, CH_3_	25.0, CH_3_	30.0, CH_3_	111.1, CH_2_	25.0, CH_3_	30.0, CH_3_	22.4, CH_3_	22.4, CH_3_	24.9, CH_3_
**27**	26.2, CH_3_	17.7, CH_3_	26.2, CH_3_	26.3, CH_3_	25.7, CH_3_	25.7, CH_3_	30.1, CH_3_	17.7, CH_3_	25.6, CH_3_	30.1, CH_3_	22.3, CH_3_	22.3, CH_3_	25.7, CH_3_
**28**	27.5, CH_3_	27.8, CH_3_	28.5, CH_3_	28.2, CH_3_	28.4, CH_3_	28.4, CH_3_	28.0, CH_3_	27.5, CH_3_	25.8, CH_3_	27.8, CH_3_	107.0, CH_2_	107.0, CH_2_	26.1, CH_3_
**29**	15.2, CH_3_	15.3, CH_3_	15.0, CH_3_	15.8, CH_3_	15.9, CH_3_	15.9, CH_3_	15.6, CH_3_	15.2, CH_3_	22.3, CH_3_	15.7, CH_3_	–	–	14.7, CH_3_
**30**	24.6, CH_3_	25.9, CH_3_	25.1, CH_3_	25.7, CH_3_	23.8, CH_3_	23.8, CH_3_	24.4, CH_3_	24.6, CH_3_	25.4, CH_3_	24.8, CH_3_	14.9, CH_3_	15.6, CH_3_	19.8, CH_3_
**32**											25.5, CH_3_	27.6, CH_3_	
**25-OMe**	50.4, CH_3_		50.4, CH_3_	50.6, CH_3_									

^a^ Measured in CDCl_3_.

^b^ In CD_3_OD.

**Table 3.** ^1^H NMR data of the isolated tirucallane (**4**–**6**), ergostane (**14**, **15**), and cycloartane (**16**) triterpenes [500 MHz, CD_3_OD, *δ* ppm (*J*  =  Hz)].

Position	4^a^	5	6	14	15	16
**1α**	1.60, m	1.52, m	1.53, m	1.43, m	1.07, m	1.28, m, 1.58, m
**1β**	2.49, m	1.84, dt (13.1, 3.4)	1.85, dt (13.1, 3.3)	1.91, m	2.92, m	
**2α**	1.76, m (2H)	1.34, m	1.34, m	1.89, m	1.78, m	1.63, m; 1.70, m
**2β**		1.69, m	1.69, m	1.58, m	1.56, m	
**3**	3.26, dd (11.1, 5.3)	3.16, dd (10.8, 5.4)	3.16, dd (10.2, 5.4)	3.05, dt (10.3, 4.9)	3.06, ddd (10.6, 10.2, 5.0)	3.22, dd (10.7, 4.4)
**4**				1.46, m	1.41, m	–
**5**	1.67, m	1.26, dd (12.0, 5.1)	1.26, m	1.51, m	1.28, m	1.31, m
**6**	2.45, m	2.23, m α	2.22, m α	0.83, m	1.45, m	0.83, m
	2.39, m	2.13, m β	2.11, m β	1.62, m	1.83, m	1.62, m
**7**	–	5.38, d (2.9)	5.38, d (4.1)	2.25, m; 2.41, m	4.30, d (3.6)	1.11, m; 1.35, m
**8**				–	–	1.56, m
**11**	4.67, t (8.2)	5.24, d (3.9)	5.24, d (4.1)	2.38, m; 2.44, m	–	1.37, m; 2.05, m
**12α**	2.31, mα	2.20, m	2.20, m	1.84, m (2H)	2.76, d (16.9)α	1.67, m (2H)
**12β**	1.87, m	2.28, dd (17.7, 5.3)	2.30, dd (17.6, 5.2)		2.43, d (16.9)	
**15α**	1.41, m	1.37, m	1.37, m	1.34, m (2H)	1.75, m	1.34, m (2H)
**15β**	2.03, m	1.99, m	2.00, m		1.96, m	
**16**	1.70, m; 2.03, m	1.70, m (2H)	1.70, m (2H)	1.71, m; 2.02, m	1.44, m; 2.08, m	1.37, m; 1.96, m
**17**	1.65, m	1.66, m	1.63, m	1.50, m	1.83, m	1.66, m
**18**	0.78, s	0.68, s	0.68, s	0.73, s	0.84, s	1.02, s
**19**	1.27, s	0.94, s	0.94, s	1.22, s	1.03, s	0.36, d (4.0); 0.58, d (4.0)
**20**	1.56, m	1.47, m	1.48, m	1.43, m	1.45, m	1.43, m
**21**	0.89 d (6.2)	0.90, d (6.4)	0.89, d (6.7)	0.98, d (6.6)	0.93, d (6.4)	0.92, d (6.6)
**22**	1.94, m; 2.39, m	1.03, m; 1.98, m	1.28, m; 1.72, m	1.15, m; 1.62, m	1.18, m; 1.63, m	1.00, m; 1.83, m
**23**	5.60, m	1.75, m	1.52, m	1.91, m	1.80, m	1.13, m
		1.13, m	1.40, m	2.15, ddd (14.0, 11.1, 4.7)	2.16, ddd (14.7, 11.5, 4.6)	1.74, m
**24**	5.42, d (16.0)	3.17, brd (8.8)	3.25, dd (10.3, 1.2)	–	–	3.17, dd (8.8, 5.5)
**25**				2.24, sept (6.9)	2.25, sept (7.1)	–
**26**	1.26, s	1.14, s	1.13, s	1.03, d (6.9)	1.04, d (7.1)	1.13, s
**27**	1.26, s	1.17, s	1.16, s	1.04, d (6.9)	1.04, d (7.1)	1.16, s
**28**	0.98, s	0.97, s	0.96, s	4.73, s 4.67, s	4.68, s 4.74, s	0.95, s
**29**	0.91, s	0.88, s	0.88, s	–		0.81, s
**30**	1.16, s	0.88, s	0.88, s	0.98, d (6.1)	1.03, d (6.3)	0.94, s
**32**				0.95, s	1.28, s	

^a^ 25-OMe**:** 3.16, s.

### Biological assays

Epimastigotes of *T. cruzi* (Y strain, ATCC 50832) were cultured by weekly passages at 28 °C in liver infusion tryptose (LIT) medium supplemented with 10% heat-inactivated fetal bovine serum (hiFBS), following previously described methods (Ioset et al. 2009). Unless otherwise specified, epimastigote cultures in the logarithmic growth phase (1–5 × 10^7^ parasites/mL) were used for the experiments. The green fluorescent protein (GFP)-overexpressing epimastigotes were generated by transfection with pTREX-n-eGFP, a gift from Rick Tarleton (Addgene plasmid # 62544; http://n2t.net/addgene:62544;RRID:Addgene_62544), as described previously. GFP-expressing epimastigotes were maintained in LIT medium supplemented with 10% hiFBS and 500 *μ*g/mL of G418.

According to the Drugs for Neglected Diseases initiative (DNDi), a promising lead compound should be active against trypanosomes at concentrations ≤10 *μ*M and exhibit at least 10-fold greater potency against *T. cruzi* than against mammalian cells (Ioset et al. 2009). The selectivity of the isolated compounds was evaluated by measuring their trypanocidal activity against epimastigotes using the Cell Proliferation Kit II (XTT) assay, following previously described procedures (Salm et al. 2021). Briefly, 1.5 × 10^5^ epimastigotes were seeded per well in 96-well plates. Compounds were applied either at a single concentration of 10 *μ*M for initial screening or across six concentrations ranging from 10 to 0.3 *μ*M for 72 h at 28 °C. Benznidazole was used as the positive control in all experiments. After incubation, plates were examined under a microscope to confirm sterility and monitor control growth. Then, 50 *μ*L of XTT and PMS (phenazine methosulfate, Sigma-Aldrich, MO, USA) solution (0.5 mg/mL XTT and 0.025 mg/mL PMS) was added to each well, and the plates were incubated at 28 °C for 2.5 h. Parasites were fixed with 50 *μ*L of methanol for 15 min before measuring absorbance at 490 nm using a Tecan plate reader. Data are expressed as percentage cell viability relative to vehicle controls or as IC_50_ values calculated using GraphPad Prism version 8.0. All experiments were performed in triplicate in at least two independent assays.

Fluorescence-activated cell sorting (FACS) quantification of parasites released from infected RAW264.7 cells (otained from ATCC, TIB-71) was performed as follows: host cells were plated in 24-well plates at densities of 40,000 and 10,000 cells/mL and allowed to adhere for 24 h. Cells were then infected at a multiplicity of infection of 10 with trypomastigotes harvested using the swim-out method described above. Noninternalized trypomastigotes were removed by washing, and fresh RPMI with 2% FBS, and the test compounds were added. All experiments, including dimethyl sulfoxide (DMSO) and benznidazole controls, were conducted in triplicate. Six days post-infection, released parasites were fixed with 4% paraformaldehyde in phosphate-buffered saline for 1 h, and the samples were subsequently analyzed by FACS (BD Biosciences), according to previously described procedures (Salm et al. 2021). Intracellular amastigotes were counted as described previously (Háznagy et al. 2024).

The cytotoxic and antiproliferative effects of compound **15** were evaluated in the human leukemia cell lines HL60 (acute promyelocytic leukemia, obtained from ATCC, CCL-240) and U937 (histiocytic lymphoma, obtained from ATCC, CRL-1593.2). Cells were cultured in RPMI-1640 medium supplemented with 10% heat-inactivated FBS, 100 U/mL penicillin, and 100 µg/mL streptomycin at 37 °C in a humidified atmosphere containing 5% CO_2_.

Cells were seeded into 96-well plates at a density of 5 × 10^4^ cells per well in 100 *μ*L of culture medium and allowed to equilibrate for several hours prior to treatment. Compound **15** was dissolved in DMSO and added to the cells at progressively increasing concentrations. The final DMSO concentration did not exceed 0.1% (v/v) in any experiment. Vincristine was used as a positive control. Cells treated with vehicle (0.1% DMSO) were used as the negative control.

After 72 h of incubation, cell viability was assessed using the MTT assay. Briefly, 10 *μ*L of MTT solution [5 mg/mL in phosphate-buffered saline (PBS)] was added to each well, and the plates were incubated for 3–4 h at 37 °C. The resulting formazan crystals were dissolved in DMSO, and the absorbance was measured at 570 nm using a microplate reader. Cell viability was expressed as a percentage relative to that of vehicle-treated control cells. IC_50_ values were determined from dose–response curves using nonlinear regression analysis in GraphPad Prism. All experiments were performed in triplicate and repeated independently at least three times.

Peripheral blood mononuclear cells (PBMCs) were isolated from human venous blood *via* density gradient centrifugation using Ficoll-Paque. Briefly, venous blood (10 mL) was collected into anticoagulant-containing tubes and diluted 1:1 with sterile PBS. The diluted blood was carefully layered onto Ficoll-Paque in a 50 mL conical tube and centrifuged at 400 × *g* for 30 min at room temperature without using the brake. Peripheral blood mononuclear cells (PBMCs) were isolated from anonymized human donor blood obtained from the Blutspendezentrum Bern. The use of these samples complies with the principles of the Declaration of Helsinki. Donors provided informed consent for the use of their blood for research purposes at the time of donation. According to local regulations, the use of fully anonymized human samples does not require additional specific ethical approval.

After centrifugation, the PBMC layer at the plasma–Ficoll interface was carefully collected and transferred to a new tube. The cells were washed twice with PBS by centrifugation at 300 × *g* for 10 min to remove platelets and residual Ficoll. The final cell pellet was resuspended in RPMI-1640 medium supplemented with 10% heat-inactivated FBS, 100 U/mL penicillin, and 100 *μ*g/mL streptomycin. Cell number and viability were assessed by trypan blue exclusion using a hemocytometer. The isolated PBMCs were then immediately used for cytotoxicity assays.

The cells were washed with PBS and resuspended in RPMI-1640 medium supplemented with 10% heat-inactivated FBS and antibiotics. PBMCs were seeded at 1 × 10^5^ cells per well in 96-well plates and treated with compound 15 at the specified concentrations. Vehicle-treated cells (0.1% DMSO) served as controls. After incubating for 72 h at 37 °C and 5% CO_2_, cell viability was assessed using the MTT assay. Briefly, MTT solution (5 mg/mL in PBS) was added to each well, and the plates were incubated for 3–4 h at 37 °C. The resulting formazan crystals were solubilized in DMSO, and their absorbance was measured at 570 nm using a microplate reader. Cell viability was expressed as a percentage of the vehicle control. Experiments were conducted in triplicate and repeated independently at least three times.

To assess the immunomodulatory effects of compound **7** in infected macrophages, TNF-*α* and IL-10 concentrations were measured in cell culture supernatants using enzyme-linked immunosorbent assay (ELISA) kits obtained from Thermo Fisher Scientific (Invitrogen), according to the manufacturer’s protocol. RAW264.7 cells were seeded in 24-well plates and infected with *T. cruzi* trypomastigotes as described above. After removing noninternalized parasites, the infected cells were treated with compound **7** at the indicated concentrations or with the vehicle control (DMSO) for 24 h. After incubation, cell culture supernatants were collected and centrifuged to remove debris. The levels of TNF-*α* and IL-10 were determined using commercially available mouse ELISA kits according to the manufacturer’s instructions. Absorbance was measured with a microplate reader at the recommended wavelength, and cytokine concentrations were calculated from standard curves generated with recombinant cytokine standards. Data are expressed as pg/mL, and all experiments were conducted in triplicate across at least three independent trials.

### Statistical evaluation

Statistical analyses were performed using GraphPad Prism version 10.0 and/or R version 4.4. Data are presented as mean ± SD of independent biological experiments, as specified in the corresponding figure legends. For experiments involving three or more treatment groups, one-way ANOVA followed by Dunnett’s multiple-comparisons test was used when individual treatments were compared with a common vehicle control. Data are presented as mean ± SD from three independent biological experiments, each performed in technical triplicate. Technical replicates were averaged within each biological experiment before statistical analysis. Concentration-response curves and IC_50_ values were obtained by nonlinear regression. A *p* value <0.05 was considered statistically significant.

## Results and discussion

The CHCl_3_ fraction of the MeOH extract from the aerial parts of *E. systyloides* was separated by multistep chromatography, yielding 16 pure compounds (**1**–**16**) (Figure 2).

Compound **1** (euphosystyloidin A) was isolated as a colorless amorphous material with an optical rotation of [*α*]_D_^26^ +19.5 (*c* 0.3, MeOH). Its molecular formula was established as C_31_H_50_O_3_ by HR-APCI-MS, with a molecular peak at *m/z* 471.3827 [M + H]^+^ (calcd for C_31_H_51_O_3_^+^: 471.3838). The ^1^H NMR and ^13^C NMR JMOD spectra indicated a triterpene structure comprising eight quaternary carbons, 6 methins, 8 methylenes, and 8 methyl groups (Tables 1 and 2). The NMR spectra exhibited resonances for one keto group (*δ*_C_ 198.3), one disubstituted olefin [*δ*_C_ 128.7, 136.9; *δ*_H_ 5.40 d (*J* = 15.9 Hz), 5.52 ddd (*J* = 15.9, 7.8, 7.3 Hz)] and one tetrasubstituted olefin (*δ*_C_ 139.1, 165.3), one oxymethine group [*δ*_C_ 78.2; *δ*_H_ 3.29 br d (*J* = 9.8 Hz)], seven tertiary methyls (*δ*_C_ 16.1, 18.8, 26.0, 26.2, 27.5, 15.2, 24.6; *δ*_H_ 0.76 s, 1.06 s, 2 × 1.25 s, 1.00 s, 0.90 s, 0.98 s) and one secondary methyl [*δ*_C_ 19.3; *δ*_H_ 0.86 d (*J* = 5.7 Hz)], and one methoxy group (*δ*_C_ 50.4; *δ*_H_ 3.15 s).

**Figure 3.**
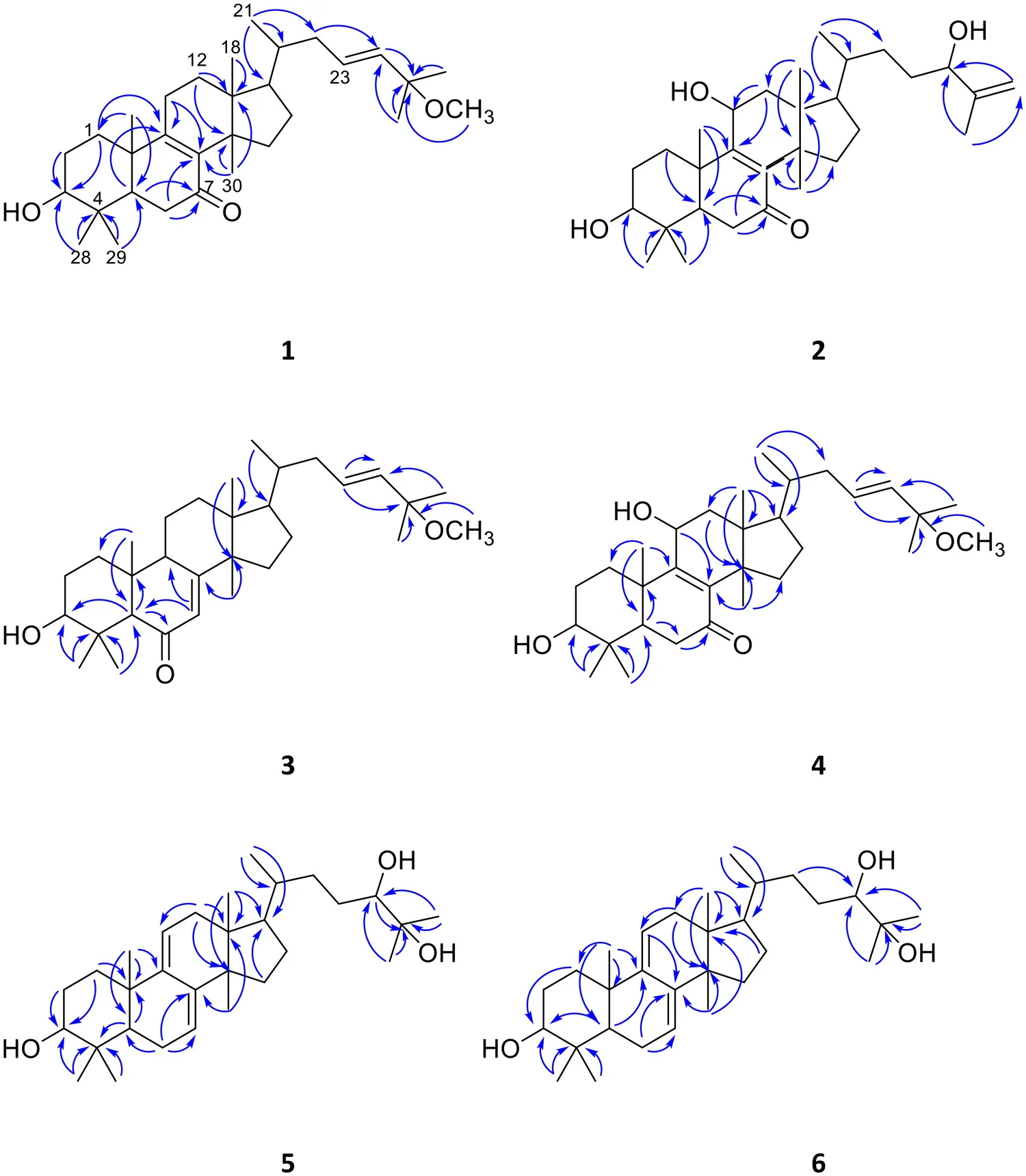
Key HMBC correlations of compounds **1**–**6** (H
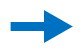
C).

**Figure 4.**
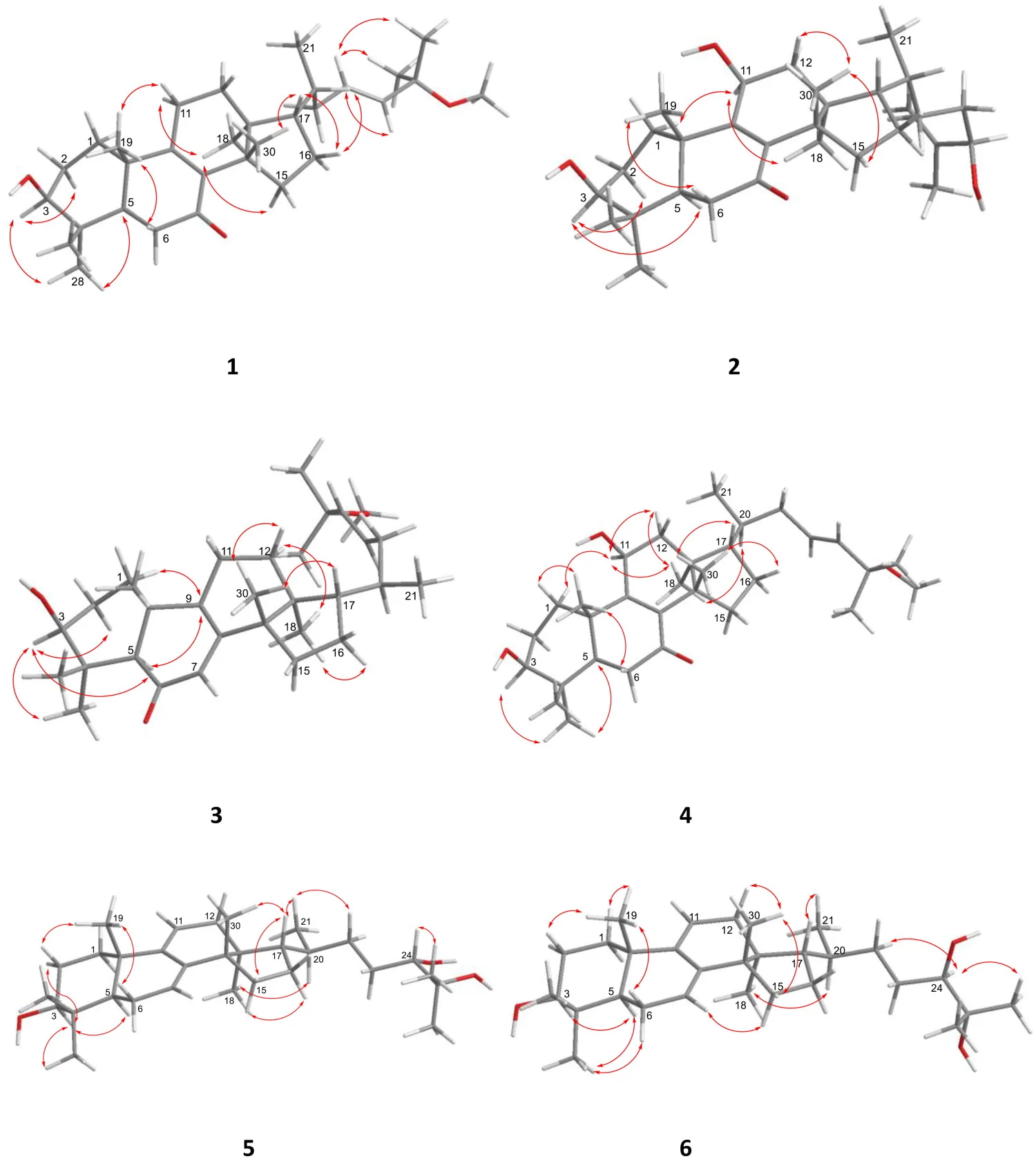
Key NOESY correlations of compounds **1**–**6** (H
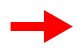
H).

Detailed analysis of the ^1^H-^1^H COSY spectrum allowed the assignment of four sequences of correlated protons: –CH_2_–CH_2_–CH– (*δ*_H_ 1.41 m, 1.86 m, 1.54 m, 1.68 m, 3.29 br d) (C-1–C-3); –CH–CH_2_– (*δ*_H_ 1.67 dd, 2.36 m, 2.44 dd) (C-5–C-6); –CH_2_–CH_2_– (*δ*_H_ 2.24 dt, 2.37 m, 1.79 m) (C-11–C-12); and –CH_2_–CH_2_–CH–CH(CH_3_)–CH_2_–CH=CH– (*δ*_H_ 1.52 m, 3.13 br t, 1.36 m, 1.94 m, 1.53 m, 1.55 m, 0.86 d, 1.86 m, 2.37 m, 5.52 ddd, 5.40 d) (C-15–C-17–C-20(C-21)–C-22–C-24). HMBC correlations of C-3 with H_2_-2, H_2_-3, H_3_-28, and H_3_-29 confirmed the presence of a hydroxy group at C-3. The 8-en-7-one structural part was confirmed by the long-range correlations of C-7 with H-5 and H_2_-6, C-8 with H_2_-6, H_2_-11, and H_3_-30, and C-9 with H-5, H_2_-1, H_2_-11, and H_3_-19. The HMBC cross-peaks between C-25 and H-24, H_3_-26, H_3_-27, and OCH_3_ established the positions of the second olefin group at C-23-C-24 and the methoxy group at C-25. Further HMBC correlations shown in Figure 3 allowed the determination of the complete planar structure of **1**. The relative configuration of the compound was established using the NOESY spectrum. Overhauser effects between H-3/H_3_-28, H_3_-28/H-5, H_3_-18/H-15*α*, H-15*β*/H_3_-30, H-16*β*/H-17, H-16*β*/H-20, H-17/H_3_-30, H_3_-18/H-11*α*, and H-11*β*/H_3_-19 (Figure 4) provided evidence for the 3*β*-hydroxy-25-methoxyeupha-8,23*E*-dien-7-one structure of **1**.

Compound **2** (euphosystyloidin B) was obtained as a white amorphous solid with an optical rotation of [*α*]_D_^26^ +0.4 (*c* 0.3, MeOH). The molecular formula of **2** was determined as C_30_H_48_O_4_ based on the molecular ion at *m/z* 455.3517 [M + H - H_2_O]^+^ (calcd for C_30_H_47_O_3_^+^: 455.3525) in the HR-APCI-MS. Evaluation of the ^1^H NMR,^13^C NMR JMOD (Tables 1 and 2), and two-dimensional NMR spectra led to the conclusion that the tetracyclic part of **2** is identical to that of euphorol B (Wang et al. 2016), while the side chain at C-17 matches that of sooneuphanone D (Gao and Aisa 2017). Accordingly, the structure of **2** was determined to be (24 *R*)-3*β*,11*β*,24-trihydroxyeupha-8,25-dien-7-one. The key HMBC and NOESY correlations confirming the structure of **2** are presented in Figures 3 and 4.

Compound **3** (euphosystyloidin C), a white amorphous solid with an optical rotation of [*α*]_D_^26^ +7.7 (*c* 0.2, MeOH), was determined by HR-APCI-MS to have the same molecular formula, C_31_H_50_O_3_, as compound **1** [*m/z* 471.3826 [M + H]^+^ (calcd for C_31_H_51_O_3_^+^: 471.3838)]. According to the ^1^H and ^13^C NMR spectra and two-dimensional NMR measurements, it contains a keto group (*δ*_C_ 200.1), two olefins [*δ*_C_ 125.2, 128.9, 136.9, 170.5; *δ*_H_ 5.70 d (*J* = 1.9 Hz), 5.51 dt (*J* = 15.6, 7.0 Hz, 5.40 d (*J* = 15.6 Hz)], one of which is at the C-23–C-24 position, a hydroxyl group at C-3 [*δ*_C_ 79.3; *δ*_H_ 3.22 br d (*J* = 11.0 Hz)], and a methoxy group at C-25 [*δ*_C-25_ 75.0; OCH_3_: *δ*_C_ 50.4; *δ*_H_ 3.15 s), similar to compound **1** (Tables 1 and 2). The two compounds differed mainly in the chemical shifts of ring B because the 7-one-8-en structure of **1** was replaced by a 6-one-7-en structure in **3**. This was indicated by the HMBC correlations of H-5 with C-6 and C-9, H-7 with C-5 and C-9, H_3_-30 with C-8, and H_3_-19 with C-5. Additional HMBC correlations support the planar structure, as shown in Figure 3. The stereochemistry of compound **3** was determined using the NOESY spectrum. Overhauser effects between H_3_-18/H-16*α*, H-16*β*/H_3_-30, H_3_-30/H-17, H-20/H-12*β*, and H-12*α*/H_3_-18 were indicative of an euphane skeleton, while NOESY cross-peaks between H-3/H-5, H-3/H-1*α*, H-1*α*/H-9, and H-9/H-5 confirmed the *α* position of H-3 and H-9.

Compound **4** (euphosystyloidin D) was obtained as a white amorphous solid with an optical rotation of [*α*]_D_^26^ −2.2 (*c* 0.2, MeOH). The molecular formula C_31_H_50_O_4_ was determined by HR-APCI-MS, which showed a quasi-molecular ion peak at *m/z* 487.3765 [M + H]^+^ (calcd for C_31_H_51_O_4_^+^: 487.3782). The 1D and 2D NMR spectra of **4** indicated the presence of eight quaternary carbons, including a keto group, six methines (two of which are olefin methines), seven methyls, including six tertiary and one secondary methyls, one methoxy group, together comprising a triterpene (Tables 2 and 3). The ^1^H and ^13^C NMR data of the tetracyclic core closely resembled those of compound **2**, while the side chain exhibited a 23-en-25-methoxy substitution pattern, as observed in compound **1**. Detailed analysis of the ^1^H–^1^H COSY, HSQC, and HMBC spectra confirmed that compound **4** has a *Δ*^8,23^-diene-7-keto-3,11-dihydroxy-25-methoxy structure. Furthermore, ROESY correlations provided insights into the stereochemical features of **4**. Specifically, Overhauser effects between H-3/H_3_-28, H_3_-28/H-5, H-11/H-12*α*, H-12*α*/H_3_-18, H_3_-30/H-17, and H_3_-18/H-20 fully supported a tirucallane-type triterpene backbone and the *β* position of the hydroxy groups at C-3 and C-11.

Compounds **5** (euphosystyloidin E) and **6** (euphosystyloidin F) were isolated as white amorphous substances with optical rotation values of [*α*]_D_^26^ −66.7 (*c* 0.1, MeOH) and [*α*]_D_^26^ −53.3 (*c* 0.2, MeOH), respectively. Compounds **5** and **6** were assigned the same molecular formula, C_30_H_50_O_3_, based on their respective HR-APCI-MS quasi-molecular peaks at *m/z* 459.3826 and 459.3817, both corresponding to [M + H]^+^ (calcd for C_30_H_51_O_3_^+^: 459.3833). Analysis of the ^1^H and ^13^C NMR data revealed that the compounds are structurally very close isomers. Careful comparison of the ^1^H-^1^H COSY, HSQC, and HMBC spectra of compounds **5** and **6** confirmed that they shared the same planar structure (Tables 2 and 3). Two trisubstituted olefin groups, indicated by the NMR signals at *δ*_C_ 116.5/116.6 (C-11), 119.9 (C-7), 142.6 (C-8), 146.6 (C-9) and *δ*_H_ 5.24 d (H-11) and 5.38 d (H-7), were assigned in both compounds to the C-7–C-8 and C-9–C-11 positions based on HMBC correlations of H-12*β*, H-5, and H_3_-19 with C-9, H-6*α* and H_3_-30 with C-8, H-12*β* with C-11, and H-6*α* with C-7. Additionally, HMBC cross-peaks of H-1*β*, H-2*β*, H_3_-28, and H_3_-29 with C-3 (*δ*_C_ 79.7/79.8), and of H_3_-26 and H_3_-27 with C-24 (*δ*_C_ 80.5/79.6) and C-25 (*δ*_C_ 79.9), indicating the positions of the hydroxy groups at C-3, C-24, and C-25, were observed in both **5** and **6**. Comparison of the NMR assignments led to the conclusion that their tetracyclic cores are identical, and that both are tirucallane triterpenes, as evidenced by the NOESY correlations of H-17/H_3_-30 and H_3_-18/H-20 for **5,** and H-17/H_3_-21 and H_3_-18/H-20 for **6**. However, their side chains at C-17 differ significantly around the chiral carbon C-24. The chemical shifts of C-24 (*δ*_C_ 80.5) and H-24 (*δ*_H_ 3.17 brd, *J* = 8.8 Hz) in compound **5** were very similar to those of (24*S*)-1*β*,3*β*,24,25-tetrahydroxy-7,9(11)-euphadiene (*δ*_C-24_ 80.7; *δ*_H-24_ 3.18 dd, *J* = 10.0 and 1.6 Hz), isolated from *Cassipourea lanceolate* (Hou et al. 2010), confirming the 24*S* configuration for **5**. Consequently, compound **6** has a 24 *R* configuration, corroborated by its chemical shifts closely matching those of ricinodol (**12**) (**6**: *δ*_C-24_ 79.6; *δ*_H-24_ 3.25 dd, *J* = 10.3 and 1.2 Hz; **12**: *δ*_C-24_ 79.5; *δ*_H-24_ 3.26 d, *J* = 10.1 Hz), which was also isolated in this experiment.

Known compounds **7**–**16** were also isolated from the chloroform extract of *E. systyloides* and identified through one- and two-dimensional NMR experiments. All compounds were obtained from this species for the first time. Our NMR investigations enabled the assignment of the ^1^H and ^13^C NMR chemical shifts for compounds **9**, **10**, **12**, and **13**–**16** in solvents different from those previously reported (Tables 1–3). Kansenonol (**7**) and 11-oxokansenonol (**9**) were first isolated from *E. kansui* and later from other *Euphorbia* species (Wang et al. 2003; Gao and Aisa 2017; Wang et al. 2022). These triterpenes induced significant cleavage arrest when tested on isolated cells from early *Xenopus laevis* embryos (Wang et al. 2003). Sooneuphanone D (**8**) was previously reported only from *E. soongrica,* and exhibited remarkable multidrug resistance reversal activity that exceeded that of the positive control, verapamil (Gao and Aisa 2017). The 24-stereoisomer of compound **8**, (+)-(24*S*)-eupha-8,25-diene-3*β*,24-diol-7-one (**10**) and (23*E*)-3*β*,25-dihydroxytirucalla-8,23-dien-7-one (**13**) were previously obtained from *E. micractina*; neither showed activity in cytotoxicity tests against the A2780 ovarian cancer cell line, HIV-1 replication assays, or protein tyrosine phosphatase 1B inhibition assays (Xu et al. 2009). The isolated compounds (23*E*)-3*β*,25-dihydroxyeuph-7,23-dien-6-one (**11**) and ricinodol E (**12**) belong to the rarely occurring 6-on-7-ene substituted euphanes (Yu et al. 2015; Warashina and Shirota 2021). The latter compound was isolated here for the second time. Yu et al. (Yu et al. 2015) reported the inhibitory activity of compound **12** against both human and mouse 11*β*-hydroxysteroid dehydrogenase type 1 enzymes (IC_50_ 0.36 ± 0.26 *μ*M and 0.84 ± 0.18 *μ*M, respectively) (Yu et al. 2015). In the current study, two ergostane triterpenes, (3*β*,4*α*,5*α*)-3-hydroxy-4,14-dimethylergosta-8,24(28)-dien-7-one (**14**) and 3*β*,7*α*-dihydroxy-4*α*,14*α*-dimethyl-5*α*-ergosta-8,24(28)-dien-11-one (**15**), and one cycloartane triterpene (24 *R*)-3*β*,24,25-trihydroxycycloartane (**16**) were also isolated (Della Greca et al. 1994; Tanaka et al. 1999, 2000).

All isolated triterpenoids were tested for their cytotoxicity in RAW264.7 macrophages, which served as host cells for *T. cruzi* infection and amastigote replication. As shown in Table 4, several triterpenes exhibited significant cytotoxicity, but overall, no selective toxicity toward *T. cruzi* epimastigotes (the insect form) was observed. Some triterpenoids were more toxic to RAW264.7 cells than to the parasite, resulting in negative selectivity indices (SI). For example, euphosystyloidin F (**6**) (IC_50_ 7.5 ± 0.1 *μ*M) was approximately 19-fold more toxic to host cells than to the parasite, whereas compound **15** (IC_50_ 3.5 ± 1.2 *μ*M) was about sixfold more toxic to host cells. A substantial difference in toxicity between host cells and epimastigotes was observed for the 24 *R*,25-dihydroxylated triterpenes **6** and **12**. Comparison of SI values for compounds **5** and **6** indicates that the 24 *R* configuration leads to higher toxicity in RAW264.7 cells. Similar high host cell toxicity was observed for the ergostane triterpenes **14** and **15**, particularly the 7-hydroxy-11-keto-substituted compound **15**.

**Table 4.** Cytotoxicity of triterpenoids on RAW264.7 macrophage host cells and *T. cruzi* epimastigotes.

Compound	IC_50_ value (*μ*M) (RAW 264.7)	IC_50_ (*μ*M) (epimastigotes)	SI IC_50_ value (RAW 264.7)/ IC_50_ (epimastigotes)
**1**	14.1 ± 1.8	28.2 ± 3.2	0.50
**2**	9.9 ± 1.4	22.4 ± 2.4	0.44
**3**	17.0 ± 2.3	29.8 ± 4.0	0.57
**4**	16.9 ± 2.1	48.5 ± 3.8	0.35
**5**	29.9 ± 3.7	40.5 ± 3.3	0.74
**6**	7.5 ± 1.2	149.3 ± 11.9	0.05
**7**	15.7 ± 1.5	37.9 ± 4.1	0.41
**8**	25.9 ± 4.0	35.8 ± 3.2	0.72
**9**	16.8 ± 3.1	39.2 ± 4.7	0.43
**10**	32.1 ± 3.8	35.4 ± 5.0	0.91
**11**	13.2 ± 2.2	46.3 ± 3.4	0.29
**12**	31.4 ± 3.9	173.6 ± 9.7	0.18
**13**	11.2 ± 0.9	>100	<0.11
**14**	7.3 ± 1.0	28.8 ± 3.2	0.26
**15**	3.5 ± 0.1	21.6 ± 3.9	0.16
**16**	12.3 ± 1.3	34.4 ± 5.0	0.36
Podophyllotoxin	0.013 ± 0.001	–	–
Benznidazol (Bnz)	–	29.4 ± 4.6	–

**Figure 5.**
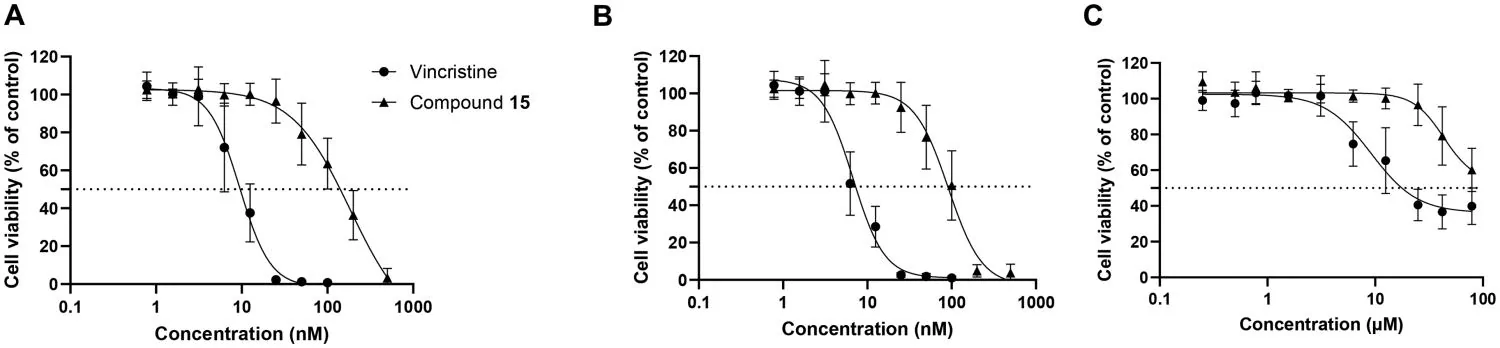
Dose–response curves showing the cytotoxic effects of compound **15** in (A) U937 and (B) HL60 leukemia cells compared with (C) peripheral blood mononuclear cells (PBMCs). Cells were incubated with increasing concentrations of compound **15** or the reference compound vincristine for 72 h. Cell viability was determined by MTT assay and expressed relative to vehicle-treated controls (0.1% DMSO). Data represent mean ± SD from four independent biological experiments, each performed in technical triplicate. Concentration–response curves were fitted by nonlinear regression.

Given the significant cytotoxic effect of compound **15**, we evaluated its cytotoxicity in human leukemia cells and peripheral blood mononuclear cells. As shown in Figure 5, compound **15** exhibited selective antileukemia activity in U937 and HL60 cells with IC_50_ values of 0.44 ± 0.21 and 0.14 ± 0.10 µM, respectively, while displaying moderate cytotoxicity toward nonproliferating PBMCs up to 100 µM. The antileukemic reference substance, vincristine, was significantly more potent, but also exhibited greater cytotoxicity against PBMCs, despite primarily targeting dividing cells. Thus, the ergostane triterpenoid **15** could serve as a starting point for the development of novel, potentially improved antileukemia molecules. To date, this class of natural products has been studied only marginally as antileukemia compounds. In a previous study, ergosta-7,22-diene-2*β*,3*α*,9*α*-triol from *Ganoderma lucidum* was reported to induce apoptosis in HL-60 human promyelocytic leukemia cells, although its potency was in the micromolar range (Lee et al. 2011). Moreover, *Antrodia camphorata* preparations enriched in ergostane/lanostane metabolites have demonstrated cytotoxicity in leukemia cells; however, in some cases, the active principles were mixtures or accompanied by lanostane compounds rather than isolated ergostanes alone (Du et al. 2012).

**Figure 6.**
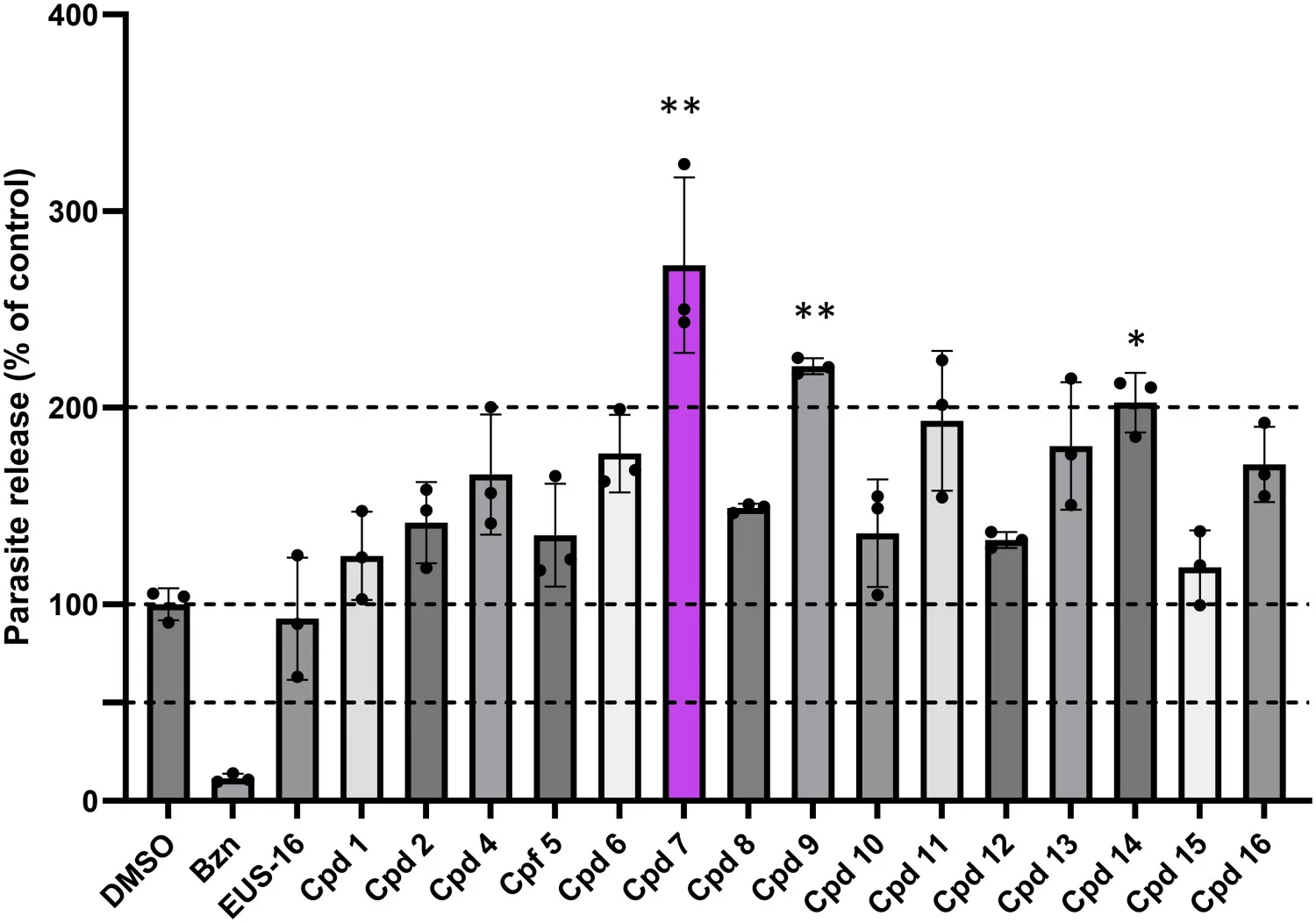
Effect of isolated triterpenoids on parasite release in *Trypanosoma cruzi*–infected RAW264.7 macrophages. Cells were infected with trypomastigotes for 24 h, washed, and subsequently treated with compounds at 1 µM as described in the methods. Released parasites correlating with amastigote replication were quantified by FACS analysis after fixation with paraformaldehyde. Data are expressed relative to vehicle-treated control cells. Several compounds increased parasite release compared to control conditions. Compound **7** significantly increased parasite release by more than twofold compared with the vehicle control. Values represent mean values ± SD from three independent experiments each performed in triplicates. Statistical significance was determined using one-way ANOVA followed by Dunnett’s multiple-comparisons test versus the DMSO control. *p < 0.05, **p < 0.01.

**Figure 7.**
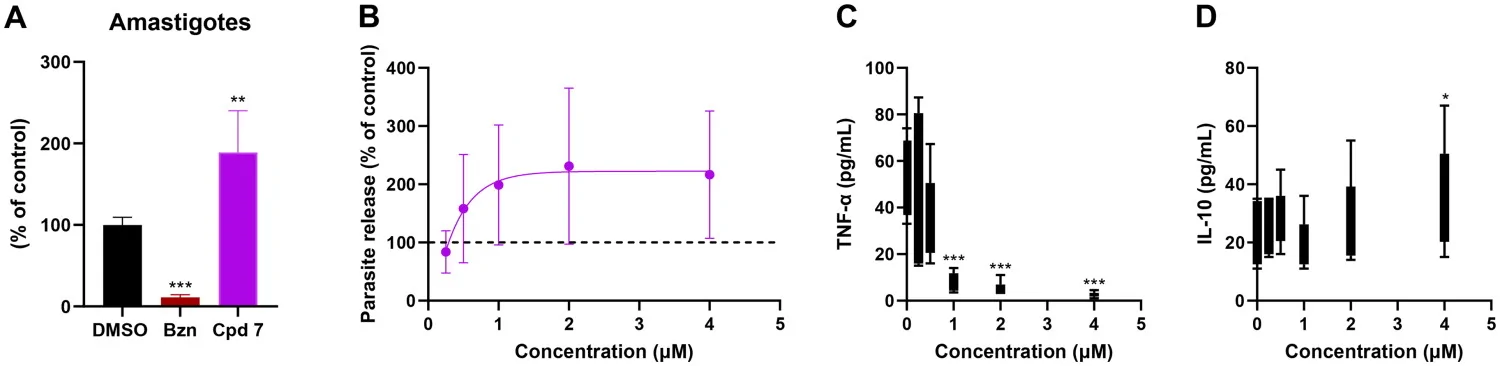
Effects of compound **7** on parasite burden and cytokine production in *T. cruzi*–infected RAW264.7 macrophages. (A) Relative number of intracellular amastigotes compared with vehicle control (DMSO). Benznidazole (bzn) (10 *μ*M) was used as a positive control. Data represent mean values ± SD from three independent experiments each performed in triplicates. Statistical significance was determined using one-way ANOVA followed by Dunnett’s multiple-comparisons test, with bzn and compound **7** compared with DMSO. **p < 0.01, ***p < 0.001. (B) Concentration-dependent increase in parasite release following treatment with compound **7**. Data represent mean ± SD from four independent biological experiments, each performed in technical triplicate. Concentration–response curves were fitted by nonlinear regression. (C) TNF-*α* levels in culture supernatants after 24 h treatment with compound **7**. (D) IL-10 levels in culture supernatants under the same conditions. Cytokines were quantified by ELISA. Data represent mean values ± SD from three independent experiments each performed in triplicates. Statistical significance was analyzed by one-way ANOVA followed by Dunnett’s multiple-comparisons test versus the DMSO control. *p < 0.05, ***p < 0.001.

Since our primary interest was to test for antichagasic effects, we evaluated selected compounds in a cellular parasite infection model to assess their effects on amastigotes, as reported previously (Salm et al. 2021), but at a moderate concentration of 1 *μ*M to avoid cytotoxicity to host cells. As shown in Figure 6, none of the triterpenes inhibited parasite infection or replication in the host cells. Unexpectedly, several compounds increased amastigote replication, especially kansenonol (**7**). Since this effect was reproducible and opposite to that of the known antichagasic compound benznidazol (Bzn; Figure 7A), we evaluated whether it was concentration-dependent. At nanomolar concentrations, there was a clear tendency for increased parasite release (Figure 7B), i.e., amastigote amounts leading to trypomastigote release. As the host cells were RAW264.7 macrophages, we speculated that the compound might influence macrophage polarization, making them either pro- or anti-inflammatory. Measuring TNF-*α* and IL-10 as markers for the proinflammatory M1 and anti-inflammatory M2 phenotypes, kansenonol (**7**) promoted the M2 polarization state, characterized by high IL-10 and low TNF-*α*. Of note, M2 macrophages are more easily infected by *T. cruzi* (Lima et al. 1997). The lack of this effect in euphosystyloidin D (**4**) suggests that the stereochemistry of the methyl group in the side chain is essential.

## Conclusions

The phytochemical investigation of *E. systyloides* yielded 16 triterpenoids, including 6 previously undescribed compounds from the euphane and tirucallane classes. These findings further highlight the structural diversity of triterpenoids within the genus *Euphorbia*, known for producing bioactive terpenoid metabolites.

Despite earlier reports of antichagasic activity in related triterpenoids (Saidu et al. 2024), none of the isolated compounds showed selective toxicity against *T. cruzi*. In several cases, the compounds were even more cytotoxic to host macrophages than to the parasite, indicating limited potential for antichagasic drug development. Instead, the biological profiling revealed other noteworthy activities.

The ergostane-type triterpenoid **15** exhibited pronounced cytotoxicity in leukemia cell lines while displaying comparatively low toxicity toward peripheral blood mononuclear cells, suggesting selectivity for proliferating cells. Although less potent than vincristine, the activity profile of compound **15** suggests that ergostane triterpenoids may represent an underexplored scaffold for antileukemic drug discovery.

Another notable finding was the unexpected increase in amastigote replication observed with kansenonol (**7**). This effect appears to be linked to immunomodulatory activity in macrophages, characterized by reduced TNF-α production and a shift toward an anti-inflammatory M2 phenotype. However, additional experiments will need to be performed to understand how kansenonol exerts this effect exactly. Because the compound was added 24 h post infection, the effect was on amastigote replication, as supported by increased amastigote counts in the altered host cell environment, most likely due to inhibition of pro-inflammatory factors. Since M2 macrophages are known to support *T. cruzi* infection, the host-directed effect likely accounts for the increased amastigote replication (Lima et al. 1997), yet additional experiments should be carried out to confirm this. Importantly, we did not observe effects on isolated epimastigote growth, which is the insect form growing outside of the host cell, supporting the indirect effect on the replicative stage. The absence of a similar effect in closely related compounds **1** and **13** suggests that subtle structural features may influence the immunomodulatory properties of these triterpenoids.

Overall, this study demonstrates that triterpenoids from *E. systyloides* primarily exert significant cytotoxic and immunomodulatory effects.

## Data Availability

The NMR data for **1 **−** 6** have been deposited in the Natural Products Magnetic Resonance Database (NP-MRD; www.np-mrd.org) with NP-MRD ID NP0333437 (**1**), NP0353952 (**2**), NP0353953 (**3**) NP0353954 (**4**), NP0353955 (**5**), NP0353956 (**6**). Other data will be made available on reasonable request from Andrea Vasas, E-mail address: vasas.andrea@szte.hu.
